# Supplementary material for: Ferritin Trajectories over Repeated Whole Blood Donations: Results from the FIND+ Study
Source: J Clin Med. 2022 Jun 21;11(13):3581. doi: 10.3390/jcm11133581 (PMC9267857; doi:10.3390/jcm11133581)
Supplement: Supplementary file 1 [file jcm-11-03581-s001.zip › jcm-1759249-supplementary.pdf]

Journal of
Clinical Medicine

MDPI

Supplementary Material: Bland Altman Plot

For logistical reasons and because of time constraints, ferritin measurements for the FIND+ study were performed in archived samples from blood donations over a period of two years. These samples had been stored at −30 °C for up to two years. To assess a potential impact of the freeze and thaw cycle and of storage time on measurement quality, we compared ferritin levels measured in archived samples to levels measured in fresh samples within 48 h after the same donation. This was achieved by linking data on ferritin measurements in archived samples for the FIND'EM study [1] to policy-derived data on ferritin measurements that had been done in fresh samples. Double ferritin measurements were available for 4427 donations. The mean difference (or bias) between both measurements was −4.4 ng/mL and lower and upper limits of agreement were −30.1 and 21.3 ng/mL. The Bland Altman plot clearly shows greater differences between measurements at higher mean ferritin levels (see Figure S1).

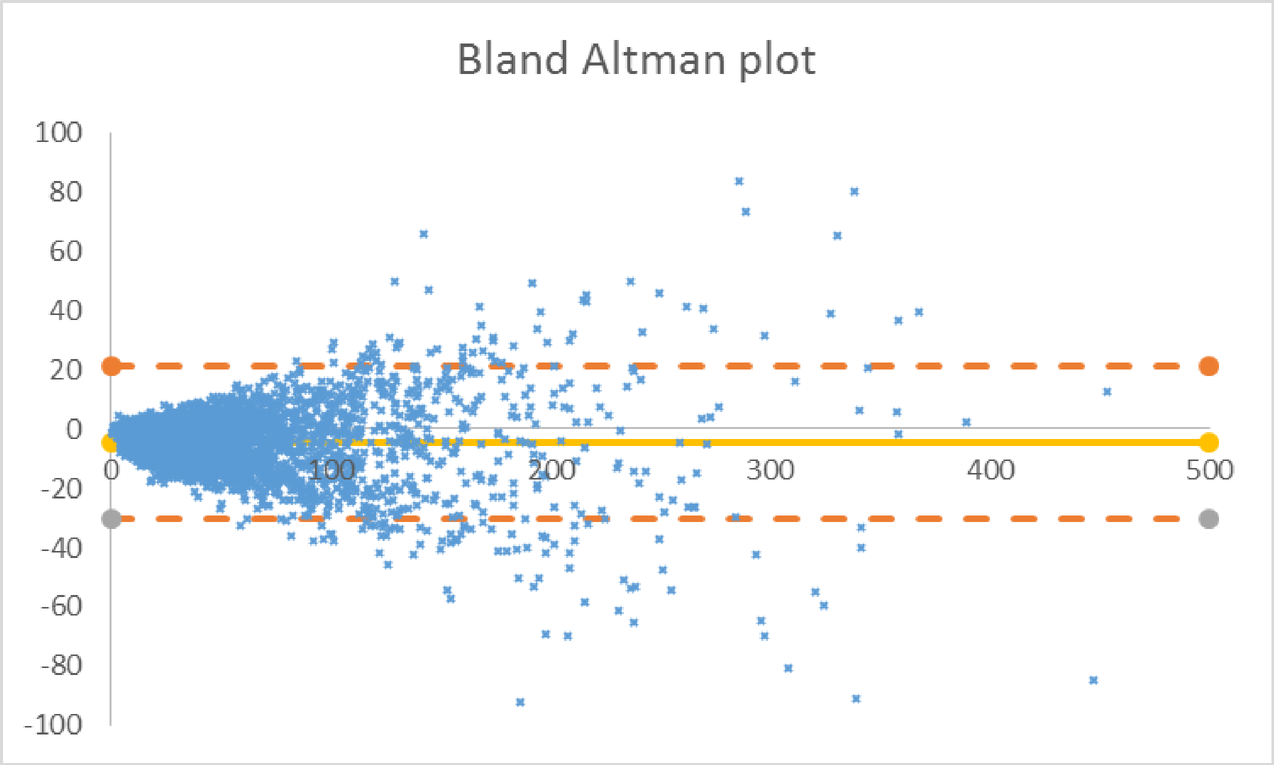

Figure S1. Bland Altman plot for ferritin measurements from archived samples (stored at −30 °C for up to 2 years) versus fresh samples, showing mean levels at the X-axis and differences at the Y-axis, showing a bias (yellow line) of −4.4 ng/mL and 95% limits of agreement (dashed orange lines) from −30.1 to 21.3 ng/mL.

Reference

1. Sweegers, M.G.; Zalpuri, S.; Quee, F.A.; Prinsze, F.J.; Hoogendijk, E.O.; Twisk, J.W.; van Weert, A.W.; de Kort, W.L.; van den Hurk, K. Ferritin measurement IN Donors-Effectiveness of iron Monitoring to diminish iron deficiency and low haemoglobin in whole blood donors (FIND'EM): Study protocol for a stepped wedge cluster randomised trial. *Trials* **2020**, *21*, 823.
